# The Differential Effect of Ego-Resiliency on the Relationship between Emotional Labor and Salivary Cortisol Level in Bank Clerks

**DOI:** 10.3390/ijerph15112576

**Published:** 2018-11-17

**Authors:** You Kyung Lim, Soo Jin Cho, Sung Min, Jeong Hoon Park, Soo Hyun Park

**Affiliations:** Department of Psychology, Yonsei University, Seoul 03722, Korea; island370@hanmail.net (Y.K.L.); sujinbin92@gmail.com (S.J.C.); annzomin@gmail.com (S.M.); jpark5664@naver.com (J.H.P.)

**Keywords:** emotional labor, salivary cortisol, ego-resiliency, bank clerks

## Abstract

Elevated stress levels in emotional laborers has been documented in a number of studies. To minimize the negative effects of stress, the need to examine potential protective factors has been highlighted. The primary purpose of this study was to investigate the differential moderating effect of ego-resiliency on the relationship between emotional labor and salivary cortisol level by comparing two groups of bank clerks deemed to experience different degree of emotional labor. Twenty-four bank clerks working in regional branch offices who provided face-to-face customer service (customer service group) and 33 administrative-duty bank clerks who work without face-to-face customer service (administrative work group) were recruited to participate in the study. Participants were asked to draw saliva into a specimen tube at an identical time during a work day and complete self-report scales measuring emotional labor and ego-resiliency. Hierarchical multiple regression analyses were conducted to examine the interaction effect of ego-resiliency on the relationship between emotional labor and salivary cortisol level by controlling for gender, age and education level as covariates. The results demonstrated that the degree of emotional labor reported by the customer service group was higher than that of the administrative work group. Furthermore, ego-resiliency moderated the relationship between emotional labor and cortisol levels in the customer service group but not in the administrative work group. The implications and limitations of this study are discussed along with suggestions for future research.

## 1. Introduction

Emotional labor refers to a type of labor wherein one must experience and/or express only particular emotions as dictated by the norms of the organization [1,2]. Thus, outward emotional expression may not be consistent and congruent with internally experienced emotions and with greater incongruence, the demand and efforts to regulate one’s emotional experience may also increase [3,4,5].

Since such processes may require non-expression of genuine emotional experiences and artificial expression of emotion, emotional labor can deplete energy and lower motivation [6,7,8]. Thus, emotional labor is significantly associated with stress, burnout and job dissatisfaction [6,9,10,11,12,13,14]. Furthermore, emotional suppression induces physiological stress responses such as the activation of the sympathetic nervous system [15,16].

Customer service bank employees represent a group of emotional laborers and oftentimes may be required to suppress genuinely experienced emotions and follow the emotional display rules required by the organization such as “service with a smile” in the course of their occupational functioning. Furthermore, with the global economic crisis in 2008, many banks underwent productive restructuring to maximize profit. The subsequent employment instability served as a stressor that negatively affected workers’ well-being. As such, interest regarding work stress and psychological and physical health of bank employees has been growing [17,18].

With increasing attention being directed towards fostering psychological adjustment of bank clerks, some researchers suggested that the effect of emotional labor might vary depending on potential moderating factors. For example, Bono and Vey reported on the results of a meta-analysis and raised the possibility that autonomy attenuated the negative effects of emotional labor on job satisfaction and burnout [9]. There has thus been a rising interest on factors that may counteract the negative effects of emotional labor [13,19], thereby providing potential means to uncover protective factors that may moderate the effects of psychological stress in emotional laborers. One such moderating factor may be ego-resiliency.

Ego-resiliency refers to the ability to exercise control and flexibility in response to situational change or stressful circumstances [20]. Individuals who score high on ego-resiliency demonstrate better psychological adjustment [21,22]. On the other hand, individuals who score lower on ego-resiliency tend to respond passively when confronted with situational demands and cope in a more rigid or problematic manner, leading to maladaptive outcomes [21,23]. Ego-resiliency is also negatively associated with perceived stress [24] and depressed mood [24,25]. Ong, Bergeman, Bisconti and Wallace demonstrated that individuals who score low on ego-resiliency experienced more difficulty in regulating negative emotions and overreacted to daily stressors [26], whereas individuals who scored higher on ego-resiliency experienced relatively more positive emotions and such positive emotions contributed to effective recovery from stressful experiences.

Self-report questionnaires may bring about a bias for responders to answer in a specific direction and there exist only a limited number of measures with standardized norms and demonstrable validity and reliability [27,28]. As such, there has also been growing interest in finding a psychophysiological index of resilience [29]. For example, cortisol is a stress hormone representing the degree of activation of the hypothalamic- pituitary-adrenal axis and is widely used as a biomarker of psychological stress [30]. Rystedt, Cropley, Devereux and Michalianou reported that in white-collar workers [31], chronic job strain significantly elevated cortisol secretion. Female care workers who experienced lower job stress showed lower cortisol secretion [32]. However, there are only a few studies that focused on the relationship between emotional labor and a stress biomarker such as cortisol.

The protective mechanism underlying ego-resiliency may be closely related to the ability to regulate negative affect. Thus, it is expected that the protective effects of ego-resiliency may differ depending on the frequency, intensity, or diversity of emotional regulation, in other words the degree of emotional labor that is required by the organization. Verifying such differential effects may offer a more effective and efficient specific intervention strategies through which psychological adjustment of emotional laborers can be secured. Therefore, we examined whether ego-resiliency differentially moderated the relationship between emotional labor and stress as measured by cortisol levels by comparing customer service bank employee group (higher emotional labor) and administrative bank employee group (lower emotional labor).

Based on such considerations, the degree of stress as measured by cortisol level may be lower in emotional laborers with higher ego-resiliency, while cortisol level may be higher in individuals with lower ego-resiliency. However, such a moderating effect of ego-resiliency may not be as evident in individuals not engaging in emotional labor.

## 2. Materials and Methods

### 2.1. Participants

The sample consisted of 57 participants (35 males and 22 females) recruited from two bank corporations in Korea. Participants were part of a larger ongoing study examining the effect of emotional labor on psychological and physical adjustment [33]. Mean age was 37.26 years (SD = 7.36) ranging from 24 to 54 years. Demographic characteristics of participants are presented in Table 1. The study protocol was approved by the Yonsei University Institutional Review Board (Approval No. 7001988-201712-SB235-08). All participants received detailed explanation about the study and gave their informed consent before they participated. All data was collected anonymously. For the purpose of collecting saliva, employees who reported having consumed any kind of food or drink were allowed to participate after 30 min from the time of intake. Only participants who had not been diagnosed with a chronic disease and not taking medication regularly were included in the study. The customer service group consisted of 24 employees working in regional branches and all participants in this group consulted customers face-to-face. The administrative work group consisted of 33 employees working in the headquarter office. Participants received a gift certificate in the amount of $14 (15,000 Korean won) as compensation for their participation.

### 2.2. Measures

Korean Emotional Labor Scale. Korean version of the Emotional Labor Scale was used to measure the perceived degree of emotional labor [34]. This scale was developed explicitly for emotional laborers in Korea. The items inquire about the nature of their labor based on current job performance situations, such as ‘I intentionally try to not express negative emotions to my customers’, ‘My feelings get hurt in the process of serving customers’. The scale consists of 24 items rated on 4-point Likert scale. Higher scores indicate higher level of emotional labor. This scale consists of 5 subscales: demands and rules of emotional regulation, overload and conflict in customer service, emotional dissonance and hurt, organizational monitoring and organizational support and protection system. The items comprising the organizational monitoring, organizational support and protection system subscales were deemed to address issues regarding the organization’s management system and were consequently not measured in the present study. Examination of its criterion validity demonstrated that all of the 5 subscales were significantly correlated with depressive symptoms (*r* = 0.22~0.46), burnout (*r* = 0.22~0.56) and quality of life (*r* = −0.34~−0.15) [34]. Cronbach’s alpha values of the original 5 subscales were all above 0.75 (0.761~0.904) [34]. The Cronbach’s coefficient was 0.83 in the present study.

Salivary cortisol levels. Saliva sample was obtained to measure cortisol levels. Saliva samples were collected in a volume of 2 mL using a commercial kit (Salimetrics, State College, PA, USA). Participants directly collected saliva in plastic vials. The vials were sealed and kept in a refrigerator. Saliva was tested using the enzyme-linked immunosorbent assay (ELISA) method and cortisol was measured in ng/mL. Level of cortisol is affected by the time of collection and typically shows highest secretion 30 min after waking up. Cortisol secretion level gradually decreases after this time and stabilizes. As such, saliva was collected at 6 pm. on a working day.

Ego-Resiliency Scale. The trait psychological resilience was measured using the Ego-Resiliency Scale [21]. Korean version of this scale that was translated by Yoo and Shim was used in this study [35]. The scale consists of 14 items such as ‘I quickly get over and recover from being startled’ and was developed to assess flexibility, curiosity, generosity and social skills. Each item is measured on a 5-point Likert scale with higher scores reflecting greater ego-resiliency. Internal consistency at the time of development was 0.76 and Cronbach’s alpha in the present study was 0.85.

### 2.3. Procedure

Following approval of the university’s Institutional Review Board, participants were given verbally instructed to not to eat or drink before saliva sampling. Sampling time was controlled at 6 pm. because cortisol level follows a circadian rhythm [36]. Before sample collection, participants were asked to wash out their mouths with water and were asked to close their mouths until saliva accumulated. Upon adequate collection, participants collected the saliva in the vial up to the 2 mL line. In cases when they experienced difficulty collecting 2 mL of saliva, they were allowed to continue collection until the amount reached the 2 mL level. After saliva collection, participants were given questionnaires and were asked to complete them within a one-week period. Questionnaires were collected by mail. Saliva samples were stored in a freezer with a set temperature of −20 °C between collection and assay. The frozen samples were post delivered to a medical research center where the samples were analyzed.

### 2.4. Data Analysis

In the present study, analyses were performed using the Statistical Program for Social Sciences (IBM SPSS Statistics for Windows, Armonk, NY, U.S.A.) Version 23 for Windows and SPSS Macro [37], applying Model 1 with 1000 bias-corrected bootstrap samples. Demographic characteristics of the sample were examined and Pearson’s correlations between variable were calculated. One-way analyses of variance (ANOVAs) were used to assess whether there were significant differences between the two groups (customer service group and administrative work group). Hierarchical multiple regression analyses were conducted to test whether ego-resiliency interacted with emotional labor to influence salivary cortisol levels and SPSS Macro was used to test the statistical significance of the relationship between emotional labor and cortisol levels at different levels of ego-resiliency in an interaction. We excluded the effect of demographic variables in testing models by controlling for gender, age and education level as covariates based on suggestions of previous studies [38,39].

## 3. Results

### 3.1. Descriptive Statistics

Descriptive statistics of each variable and the mean difference between employees in regional branches (customer service group) and those in headquarters (administrative work group) are presented in Table 2.

As expected, the degree of emotional labor was significantly higher in the customer service group (*M* = 48.25, *SD* = 5.41) compared to the administrative group (*M* = 44.52, *SD* = 5.66) (*p* < 0.05). More specifically, demands and rules of emotion regulation (*p* < 0.05), overload and conflict in customer service (*p* < 0.01) emotional labor subscales were significantly higher in the customer service group than in the administrative work group. Cortisol levels and degree of ego-resiliency were not significantly different between the two groups. In addition, statistically significant correlations among the variables were not found (Table 3).

### 3.2. The Moderating Role of Ego-Resiliency

We conducted hierarchical multiple regression analyses as proposed by Aiken and West for each group to test whether the strength of the effect of emotional labor on salivary cortisol level depended on the level of ego-resiliency [40]. Prior to analysis of the moderating effect, the independent variable and moderator variable were all centered around the mean to reduce multicollinearity. Results of testing the moderating effect for each group (customer service group and administrative work group) are presented in Table 4 and Table 5, respectively. To test whether ego-resiliency interacted with emotional labor to predict cortisol levels, we entered age and education in the first block, then emotional labor and ego-resiliency as mean-centered predictor variables and the product from these mean-centered predictors to estimate the interaction term (emotional labor × ego-resiliency).

**Figure 1:**
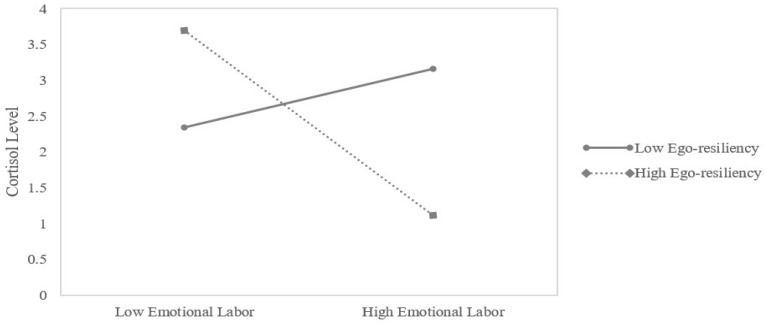
Interaction effect of emotional labor and ego-resiliency on change in cortisol levels.

In the customer service group, the interaction effect of emotional labor and ego-resiliency on cortisol levels was significant (Δ*R*^2^ = 0.265, *p* = 0.057), with the entire model accounting for 26% of the variance in cortisol levels. It demonstrated that the strength of the effect of emotional labor on cortisol levels changed depending on the level of ego-resiliency. However, a significant interaction effect of emotional labor and ego-resiliency on cortisol levels was not found in the administrative work group. To interpret the moderating effect in the customer service group, we obtained predicted values for the cortisol levels by substituting regression coefficients in the multiple regression equation and derived regression equations for each level of ego-resiliency [40,41]. The result is shown in Figure 1. In the case of high ego-resiliency, cortisol levels decreased as degree of emotional labor increased. However, at low ego-resiliency, cortisol levels slightly increased as emotional labor increased.

Meanwhile, as ego-resiliency was a continuous variable, we needed to determine the specific condition wherein the moderating effect occurred. To this end, we followed the recommendations of Aiken and West [40] who suggested the need to examine the interaction effect of the moderator at specific values (e.g., mean, mean ± 1 *SD*) and then test the statistical significance of the slopes. Hence, we tested the significance of the slopes of the simple regression lines representing the relationship between emotional labor and cortisol levels at the mean and mean ± 1 *SD* values of ego-resiliency using SPSS Macro [37]. The simple slope of emotional labor was significant for high ego-resilient bank clerks, *B* = −0.239, *t*(24) = −2.738, *p* < 0.05 but not for low ego-resilient bank clerks, *B* = 0.076, *t*(24) = 0.968, *ns* (Figure 1). Thus, high ego-resilient bank clerks (mean + 1 *SD*) showed decrease in salivary cortisol level with greater emotional labor, whereas low ego-resilient bank clerks did not exhibit this association.

## 4. Discussion

In the present study, we investigated the degree of emotional labor and salivary cortisol level, a physiological marker of stress in bank clerks. Furthermore, we examined whether ego-resiliency differentially moderated this relationship by comparing salivary cortisol levels of two groups of bank employees expected to report different degrees of emotional labor.

First, the results showed a significant difference in degree of emotional labor between the customer service and administrative work groups, with bank clerks in the customer service group who provides face-to-face customer service reporting higher degree of emotional labor. Moreover, bank clerks providing face-to-face customer service reported significantly higher degree of demands and rules of emotion regulation, overload and conflict in customer service, two subscales of the emotional labor scale. Our results are consistent with prior research (e.g., [3,4]). Also, emotional dissonance and hurt level were not significantly different between the customer service group and administrative group. This result might be related to the nature of the support system of the bank organization. Bank clerks in Korea reported the highest perceived organizational support and protection system compared to other occupation clusters [34]. Such perceived organizational support may operate as a protective factor in assisting bank clerks to adjust to the impact of emotional labor on job satisfaction and performance [42].

Interestingly, we found that ego-resiliency moderated the relationship between emotional labor and the level of salivary cortisol only in the customer service group. When ego-resiliency was high, the level of cortisol decreased even if the degree of emotional labor was high. This result is consistent with prior research which indicated that individuals with high ego-resiliency can ‘bounce back’ effectively from a stressor by experiencing more positive emotion in the stressful situation and by perceiving less stress compared to people with low ego-resiliency [24,26,43,44]. In contrast, when ego-resiliency was low, the degree of emotional labor did not predict salivary cortisol level. Individuals with low ego-resiliency may experience particular vulnerability in times of added stress. The finding that in individuals diagnosed with posttraumatic stress disorder (PTSD), a condition characterized by ongoing maladaptive responses following a significant trauma, salivary cortisol at baseline demonstrated particular inconsistencies [45]. Another prospective study exploring the cortisol-resilience connection reported that salivary cortisol response to distressing video in low-resilient police officers was significantly blunted [46]. Thus, it is possible that individuals with low ego-resiliency may demonstrate blunted cortisol level secondary to chronic stress regardless of the actual degree of emotional labor. For example, when a particular situation and subsequent outcomes are perceived to be unpredictable and uncontrollable, HPA axis may demonstrate particularly strong response characteristics [47]. Studies have also suggested that whereas small to moderate increases in cortisol may actually promote adaptive functioning, frequent and prolonged exposure to stressors may negatively affect functioning and adjustment (e.g., [48]).

In order to further explain such a differential interaction effect, the psychological nature of ego-resiliency must be discussed. High ego-resilient individuals are able to exercise flexible self-regulation, whereas low ego-resilient individuals tend to be rigid in their attempts to self-regulate [21]. Resilience has been associated with successful recovery from negative events. For example, individuals high in extraversion and emotional stability, two traits that have been associated with resilience demonstrated quicker affective recovery after viewing a negative affect arousing video [25,49]. Thus, this capacity for recovery from negative stressful experiences may be one facet through which resilient individuals are able to maintain stability in times of adversity. In this manner, even if resilient individuals experience expected degree of negative emotions and physiological indices of distress in response to stressful situations [44], they may return to homeostasis more efficiently.

The finding that the cortisol level across the customer service and administrative group was not significantly different may be due to the differential moderating effect of ego-resiliency. In other words, although emotional labor creates a stressful environment, ego-resilient workers may experience less stress because they adapt to the environment more flexibly. Thus, ego-resiliency can be an important psychological resource for emotional laborers and intervention to improve ego-resiliency may be clinically useful [50]. In a meta-analysis of interventions to enhance resilience, cognitive behavioral therapy (CBT)-based and mindfulness-based interventions, or combination of CBT and mindfulness techniques were found to be particularly effective in strengthening resilience [51].

The present study has a number of limitations and suggestions for future studies are needed. First, the moderating effect of ego-resiliency in the relationship between emotional labor and other outcome measures such as mood symptoms may provide further evidence for the role of ego-resiliency. Furthermore, the present study used only cortisol as a measure of stress. In future studies, a more diverse method of assessing stress (e.g., blood pressure, self-report measures) may strengthen the conclusions regarding emotional labor and resulting stress. Lastly, this study focused on an intraindividual variable, ego-resiliency, as a potential protective factor. However, since emotional labor takes place in the work environment, environmental and organizational aspects (e.g., perceived support of organization) should also be considered in addition to such intraindividual factors.

## 5. Conclusions

This study sought to investigate whether the degree of ego-resiliency differentially affects the relationship between emotional labor and salivary cortisol level in two groups of bank clerks deemed to experience different levels of emotional labor. The results showed a significant difference in degree of emotional labor between the customer service and administrative work groups, with bank clerks in the customer service group who provides face-to-face customer service reporting higher degree of emotional labor. The study also provided support for the moderated effect of ego-resiliency, that when ego-resiliency was high, the level of cortisol decreased even if the degree of emotional labor was high in the customer service bank clerk group but not in the administrative work group. It suggested that ego-resiliency can make individuals ‘bounce back’ effectively from a stressor and also be an important psychological resource for emotional laborers.

## Figures and Tables

**Table 1:** Demographic characteristics of participants (*N* = 57).

Variables	*n*	Frequency (%)
Gender		
Male	35	61.4
Female	22	38.6
Age		
20–29	6	10.5
30–39	33	57.9
40–49	14	24.6
50–59	4	7.0
Job Position		
Staff	13	22.8
Assistant manager	12	21.1
Manager	26	45.6
Department manager	6	10.5
Year(s) in Employment		
Under 4 years	11	19.3
5–9 years	16	28.1
10–19 years	18	31.6
over 20 years	12	21.0
Employment Status		
Permanent	52	91.2
Temporary	5	8.8
Salary (thousand Korean won)		
1000–1999	2	3.5
2000–2999	5	8.8
3000–3999	8	14.0
4000–4999	12	21.1
Over 5000	30	52.6
Work Hour Per Day		
Under 8 h	1	1.8
Around 8 h	3	5.3
Over 8 h	53	93.0
Highest Education		
High school	8	14.1
Community college	4	7.0
4-year university	41	71.9
Graduate school	4	7.0

**Table 2:** Means (standard deviations) of the Korean Emotional Labor Scale, salivary cortisol level and ego-resiliency scale.

	Customer Service Group (*n* = 24)	Administrative Work Group (*n* = 33)	*F*	*p*-Value
Emotional Labor	48.25 (5.41)	44.52 (5.66)	6.28 *	0.015
Demands and rules of emotional regulation	17.83 (1.97)	16.70 (1.93)	4.73 *	0.034
Overload and conflict in customer service	12.67 (1.99)	10.85 (2.64)	8.056 **	0.006
Emotional dissonance and hurt	17.75 (3.27)	16.97 (3.03)	0.863	0.357
Salivary Cortisol Level	2.58 (1.29)	2.09 (1.61)	1.551	0.218
Ego-Resiliency	45.96 (7.57)	44.91 (7.37)	0.275	0.602

* *p* < 0.05, ** *p* < 0.01.

**Table 3:** Correlations between study variables.

	1. Emotional Labor	2. Salivary Cortisol Level	3. Ego-Resiliency
1. Emotional labor	-		
2. Salivary cortisol levels	−0.094	-	
3. Ego-resiliency	0.210	0.044	-

**Table 4:** Results of hierarchical regression analysis predicting salivary cortisol level from emotional labor with ego-resiliency as the moderator variable in the customer service group.

Step and Variable	*B*	*SE B*	95% CI	*β*	*R* ^2^	Δ*R*^2^
Step 1					0.223	0.149
Age	0.066	0.029	0.005, 0.127	0.463 *		
Education	0.473	0.267	−0.083, 1.028	0.366		
Step 2					0.229	0.067
Age	0.064	0.034	−0.006, 0.135	0.45		
Education	0.441	0.298	−0.182, 1.065	0.342		
Emotional labor (A)	0.019	0.05	−0.085, 0.123	0.08		
Ego-resiliency (B)	−0.002	0.038	−0.081, 0.76	−0.013		
Step 3					0.49	0.348 *
Age	0.078	0.029	0.018, 0.138	0.548 *		
Education	0.441	0.249	−0.082, 0.964	0.341		
Emotional labor (A)	0.069	0.045	−0.025, 0.163	0.288		
Ego-resiliency (B)	−0.022	0.032	−0.089, 0.046	−0.128		
(A) × (B)	−0.021	0.007	−0.035, −0.006	−0.578 **		

*Note*. CI = confidence interval, * *p* < 0.05, *p* < 0.01.

**Table 5:** Results of hierarchical regression analysis predicting salivary cortisol level from emotional labor with ego-resiliency as the moderator variable in the administrative work group.

Step and Variable	*B*	*SE B*	95% CI	*β*	*R* ^2^	Δ*R*^2^
Step 1					0.079	0.017
Age	−0.088	0.055	−0.201, 0.024	−0.318		
Education	0.593	0.812	-1.066, 2.252	0.145		
Step 2					0.122	−0.004
Age	−0.104	0.061	−0.229, 0.022	−0.375		
Education	1.082	0.922	−0.807, 2.971	0.265		
Emotional labor (A)	0.036	0.061	−0.089, 0.160	0.125		
Ego-resiliency (B)	0.035	0.045	−0.058, 0.127	0.159		
Step 3					0.17	0.017
Age	−0.124	0.063	−0.252, 0.005	−0.447		
Education	0.831	0.935	−1.087, 2.748	0.203		
Emotional labor (A)	0.003	0.065	−0.130, 0.137	0.012		
Ego-resiliency (B)	0.063	0.05	−0.040, 0.165	0.286		
(A) × (B)	−0.01	0.008	−0.026, 0.006	−0.263		

*Note*. CI = confidence interval.
